# Study protocol: a cluster-randomized trial implementing Sustained Patient-centered Alcohol-related Care (SPARC trial)

**DOI:** 10.1186/s13012-018-0795-9

**Published:** 2018-08-06

**Authors:** Joseph E. Glass, Jennifer F. Bobb, Amy K. Lee, Julie E. Richards, Gwen T. Lapham, Evette Ludman, Carol Achtmeyer, Ryan M. Caldeiro, Rebecca Parrish, Emily C. Williams, Paula Lozano, Katharine A. Bradley

**Affiliations:** 1 Kaiser Permanente Washington Health Research Institute, 1730 Minor Ave, Suite 1600, Seattle, WA 98101 USA; 2 Department of Psychiatry and Behavioral Sciences, School of Medicine, University of Washington, Seattle, WA USA; 3 Department of Health Services, University of Washington, Seattle, WA USA; 4 VA Puget Sound Health Care System, Center of Excellence in Substance Abuse Treatment and Education, Seattle, WA USA; 5 Behavioral Health Services Department, Kaiser Permanente Washington, Seattle, USA; 6 VA Puget Sound, Health Services Research and Development Center of Innovation for Veteran-Centered and Value-Driven Care, Seattle, WA USA

**Keywords:** Alcohol drinking, Prevention, Alcohol use disorders, Primary care, implementation, stepped-wedge, pragmatic trial

## Abstract

**Background:**

Experts recommend that alcohol-related care be integrated into primary care (PC) to improve prevention and treatment of unhealthy alcohol use. However, few healthcare systems offer such integrated care. To address this gap, implementation researchers and clinical leaders at Kaiser Permanente Washington (KPWA) partnered to design a high-quality program of evidence-based care for unhealthy alcohol use: the Sustained Patient-centered Alcohol-related Care (SPARC) program. SPARC implements systems of clinical care designed to increase both prevention and treatment of unhealthy alcohol use. This clinical care for unhealthy alcohol use was implemented using three strategies: electronic health record (EHR) decision support, performance monitoring and feedback, and front-line support from external practice coaches with expertise in alcohol-related care (“SPARC implementation intervention” hereafter).

The purpose of this report is to describe the protocol of the SPARC trial, a pragmatic, cluster-randomized, stepped-wedge implementation trial to evaluate whether the SPARC implementation intervention increased alcohol screening and brief alcohol counseling (so-called brief interventions), and diagnosis and treatment of alcohol use disorders (AUDs) in 22 KPWA PC clinics.

**Methods/Design:**

The SPARC trial sample includes all adult patients who had a visit to any of the 22 primary care sites in the trial during the study period (January 1, 2015–July 31, 2018). The 22 sites were randomized to implement the SPARC program on different dates (in seven waves, approximately every 4 months). Primary outcomes are the proportion of patients with PC visits who (1) screen positive for unhealthy alcohol use and have documented brief interventions and (2) have a newly recognized AUD and subsequently initiate and engage in alcohol-related care. Main analyses compare the rates of these primary outcomes in the pre- and post-implementation periods, following recommended approaches for analyzing stepped-wedge trials. Qualitative analyses assess barriers and facilitators to implementation and required adaptations of implementation strategies.

**Discussion:**

The SPARC trial is the first study to our knowledge to use an experimental design to test whether practice coaches with expertise in alcohol-related care, along with EHR clinical decision support and performance monitoring and feedback to sites, increase both preventive care—alcohol screening and brief intervention—as well as diagnosis and treatment of AUDs.

**Trial registration:**

The trial is registered at ClinicalTrials.Gov: NCT02675777. Registered February 5, 2016, https://clinicaltrials.gov/ct2/show/NCT02675777.

**Electronic supplementary material:**

The online version of this article (10.1186/s13012-018-0795-9) contains supplementary material, which is available to authorized users.

## Background

Unhealthy alcohol use, a common cause of death and disability [1], includes a spectrum from risky drinking to alcohol use disorders (AUDs) [2, 3]. Risky drinking—drinking above recommended limits [4]—can lead to the development of AUDs and increases risk of a number of other health problems such as trauma, cirrhosis, cancer, and poor management of other chronic diseases [5–10]. Unhealthy alcohol use is common—over 25% of US adults report risky drinking [11], and 13.9% have AUDs [12]. Unhealthy alcohol use is often not recognized by medical providers [13], and most people with AUDs never receive treatment [12, 14].

Evidence-based care for unhealthy alcohol use includes both prevention and treatment**.** The US Preventive Services Task Force recommends routine alcohol screening and brief alcohol counseling for patients who screen positive for risky drinking (screening and brief intervention, SBI) [2, 3]. The National Commission on Prevention Priorities ranks alcohol SBI for unhealthy alcohol use as the third highest prevention priority for US adults [15]. For AUD treatment, systematic reviews and evidence-based guidelines support several treatment options, including three US Food and Drug Administration-approved medications [16, 17], counseling (motivational enhancement therapy, cognitive behavioral therapy, and couples counseling) [18], and specialty alcohol treatment [16–27].

Most health systems do not provide high-quality alcohol-related care. Most have not implemented routine SBI, and patients rarely are offered evidence-based prevention or treatment for unhealthy alcohol use. In a World Health Organization project implementing SBI across 10 nations, healthcare systems screened 2–26% of patients across countries, and rates of brief interventions among patients with unhealthy alcohol use were so low that 10% was defined as a “high” rate of brief intervention [28]. A 2011 systematic review on implementation of preventive care for unhealthy alcohol use [29] found that no healthcare system had successfully implemented sustained high-quality SBI. Receipt of effective treatments for AUD, including pharmacotherapy and specialty treatment, is also rare [30–34]. An important study found that the quality of US medical care for AUDs was poorer compared to any other common chronic disease [35], and this has not changed in the years since [36].

Efforts to implement improved alcohol-related preventive care have had both successes and challenges. One successful implementation of SBI—a preventive intervention—in healthcare settings was integration at more than 900 Veterans Affairs (VA) clinical sites nationwide [37–39]. Embedded VA investigators partnered with VA leaders to implement SBI using two strategies that addressed many issues important in dissemination and implementation: [40] (1) performance monitoring with SBI quality indicators and feedback to sites and (2) dissemination of electronic health record (EHR) decision support for screening and brief intervention [39]. Technical assistance and knowledge transfer was supported by a nationally disseminated technical manual and training materials and was performed by local leaders [41]. This process resulted in sustained high rates of documented screening (> 90%) and brief intervention (78%) across 21 VA networks [42]. Although implementation of alcohol SBI in the VA increased patient report of receiving alcohol-related advice [43], it also had limitations. The VA implementation strategies resulted in variable-quality screening [44, 45], incomplete understanding and “ownership” of preventive alcohol-related care among front-line primary care (PC) staff [46], and unclear benefits in reducing consumption among patients [47, 48]. Moreover, biased denominators may have impacted performance monitoring [45, 49], and incentives to document brief intervention may have led to increased EHR documentation of brief intervention that was already occurring [50].

Little research has addressed how to improve diagnosis and treatment of AUDs among PC patients, but most efforts focus on “referral to” or “linkage to” treatment as the only option for patients with AUDs [13, 51]. Since the COMBINE trial showed that medications could benefit patients even without specialized treatment [52], increased focus has been placed on management of AUDs in PC, often with medications [16, 17, 53]. One approach to increasing diagnosis and treatment of AUDs in PC patients is to use brief standardized measures [54] based on the *Diagnostic and Statistical Manual* (DSM) to identify AUDs [13] and link patients with care managers [17, 18, 53, 55]. Increasing focus has also been placed on shared decision-making and management of AUDs [17, 18, 53, 55–59]. These approaches to improving clinical care for AUDs have been integrated into a “Behavioral Health Lab” to support PC [60], but no previous research to our knowledge has tested implementation strategies to integrate routine assessment for AUDs and shared decision-making about treatment options into PC as a means to improve AUD diagnosis and treatment.

### The Sustained Patient-centered Alcohol-related Care (SPARC) trial

**Fig. 1:**
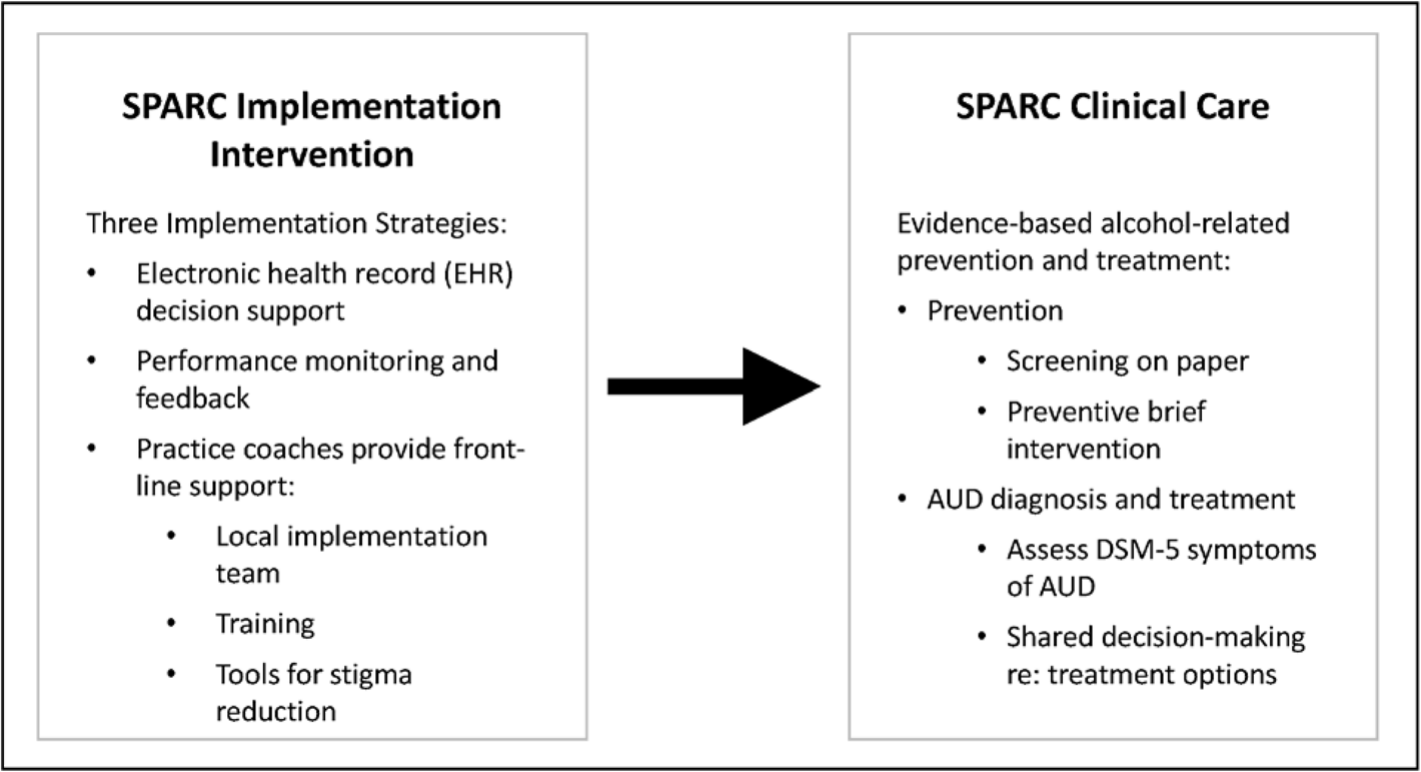
The SPARC trial: SPARC implementation intervention and alcohol-related clinical care. The SPARC implementation intervention is designed to implement improved alcohol-related clinical care including preventive screening and brief intervention for unhealthy alcohol use and increased AUD diagnosis and treatment

The Sustained Patient-centered Alcohol-related Care (SPARC) trial is testing implementation strategies to improve clinical care for unhealthy alcohol use (Fig. 1). To address gaps in preventive clinical care, the SPARC implementation intervention was designed to implement annual alcohol screening followed by brief intervention. To address gaps in clinical care of AUD (i.e., diagnosis and treatment), the SPARC implementation intervention was designed to implement routine assessment of DSM-5 AUD symptoms among PC patients with high-risk drinking and shared decision-making about evidence-based treatment options for those with active AUDs (e.g., medications and/or counseling in PC, as well as assistance accessing other AUD treatments). This alcohol-related care is referred to as “SPARC Clinical Care” hereafter (Fig. 1).

The SPARC implementation intervention includes three strategies designed to support sustained delivery of the above SPARC clinical care: EHR clinical decision support; performance monitoring and feedback; and front-line support from practice coaches with expertise in alcohol-related care (Fig. 1). These strategies built on lessons learned from the VA [37–39, 61], and were refined in a pilot study in three Kaiser Permanente Washington (KPWA) clinics in 2015 [62].

This report describes the protocol for the SPARC trial, a pragmatic, cluster-randomized, stepped-wedge trial testing the SPARC implementation intervention in 22 PC clinics of KPWA. The trial is pragmatic because front-line clinical teams implement all changes in clinical care. A cluster-randomized trial is appropriate because the implementation intervention is conducted at the clinic level. All outcomes are evaluated at the clinic level. A stepped-wedge design, with sites randomly assigned to seven staggered waves, was selected because all clinics needed to receive the SPARC implementation intervention. The objectives of the SPARC trial are to test whether the multi-faceted approach to implementation increases the proportion of PC patients who:

1. Screen positive for unhealthy alcohol use and have documented brief interventions, and
2. Have AUDs identified and subsequently initiate and engage in treatment for AUDs.

## Methods/design

### Setting

The trial is conducted in the 22 PC clinics of KPWA, which includes all PC clinics that did not participate in the three-clinic SPARC pilot study [62]. Prior to SPARC implementation, the health system had no population-based preventive SBI (e.g., when measured at three clinics, 8.9% of patients were screened for unhealthy alcohol use) [62], and of an estimated 381,550 total patients who received care at KPWA in 2014, only an estimated 0.04% per year were engaged in AUD care based on the International Classification of Diseases codes, 9th edition (ICD-9) used by the National Committee for Quality Assurance (NCQA) Healthcare Effectiveness Data and Information Set (HEDIS) measures for Alcohol and Drug Use Disorders.

### SPARC intervention

#### Context

##### Behavioral health integration added to SPARC

At the time the intervention was designed, SPARC clinical care was to be implemented alone to address unhealthy alcohol use. However, at the request of KPWA clinical leaders, parallel care for depression, suicidality, cannabis use, and other drug use was implemented at the same time and supported by the study, because the health system had no population-based screening and systematic follow-up for these conditions [62]. Thus, while this report focuses on alcohol-related care, which is the focus of the SPARC trial, the trial is evaluating implementation of alcohol-related care in the context of simultaneous implementation of a wholistic Behavioral Health Integration program. Table 1 shows the parallel tools and services for each condition.

**Table 1:** Clinical care implemented in the 22 PC clinics as part of behavioral health integration

Condition	Screen	Assess	Manage
SPARC clinical care			
Unhealthy alcohol use	AUDIT-C [84, 85]	DSM-5 AUD Symptom Checklist [59]	• Preventive brief intervention • Shared decision-making: AUD treatment options • AUD medications as indicated • Warm handoffs to LICSWs
Other Behavioral Health Integration implemented at same time supported by the SPARC trial			
Depression and suicidality	PHQ-2 [86]	PHQ-9 [87] and CSSRS [88]	• Shared decision-making: depression treatment options • Depression medications as indicated • Crisis response plan • Warm handoffs to LICSWs
Cannabis use	Single item [89, 90]	DSM-5 DUD Symptom Checklist [59]	• Shared decision-making about treatment options • Warm handoffs to LICSWs
Drug use	Single item [91]	DSM-5 DUD Symptom Checklist [59]	• Shared decision-making about treatment options • Prescribe or refer for medications for opioid use disorder • Warm handoffs to LICSWs

*AUDIT-C* Alcohol Use Disorders Identification Test-Consumption Questions; *DSM-5*, Diagnostic and Statistical Manual, 5th edition; *LICSWs*, Licensed Independent Clinical Social Workers; *PHQ-2*, two-item Patient Health Questionnaire depression screen; *PHQ-9*, nine-item Patient Health Questionnaire depression screen; *CSSRS*, Columbia Suicide Severity Rating Scale; *DUD*, drug use disorder

##### Structure of the research-operations partnership

The trial is a partnership, begun in 2014, between researchers at Kaiser Permanente Washington Health Research Institute (KPWHRI) and KPWA clinical leaders in Behavioral Health Services. Details about organization of the research-operations partnership are included in an additional file [see Additional file 1].

##### Health system addition of integrated behavioral health clinicians trained in managing substance use disorders

The initial design of the SPARC intervention did not include integrated behavioral health clinicians because they did not exist in KPWA. In the year prior to the SPARC trial, KPWA leaders decided to shift the role of licensed independent clinical social workers (LICSWs) in PC from that of medical social workers to integrated behavioral health clinicians. Details about trainings and an EHR registry used to facilitate this shift are described in an additional file [see Additional file 1].

#### The three SPARC implementation strategies

**Fig. 2:**
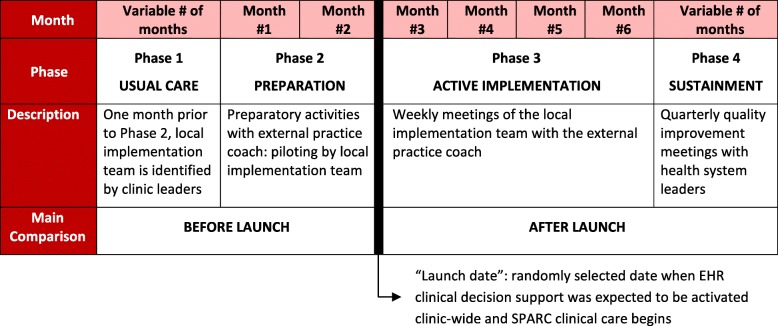
Schematic of each clinics’ four phases of the SPARC trial

The SPARC implementation intervention is a multicomponent intervention that builds on strategies known to be effective [63]. Two strategies were effective in implementing SBI in VA, and a third was added to address barriers to adoption and implementation highlighted in the VA [41, 44–46]. The SPARC implementation intervention integrates (1) EHR decision support [64], (2) performance monitoring and feedback [65], and (3) front-line support by practice coaches to address limitations of the VA approach [63, 66]. Specifically, front-line support of PC teams by practice coaches address PC adopters’ needs including overcoming stigma, improving knowledge about evidence-based alcohol-related care, and increasing staff ownership about the value of providing their patients alcohol-related care. Practice coaches work with each clinic for about 2 months before implementation and about 4 months after (Fig. 2). Throughout the trial, weekly formative evaluation meetings are used to identify refinements to the implementation intervention, if needed, as well as barriers and facilitators to address and capitalize on, respectively.

1. *EHR decision support*. EHR decision support was developed to guide screening for, assessing, and managing unhealthy alcohol use (Table 1) [64]. EHR tools are described in detail in an additional file [see Additional file 1]). Briefly, EHR prompts alert medical assistants (MAs) or other staff who room patients (MAs hereafter) to give patients a seven-item paper behavioral health screen, which includes the three-item Alcohol Use Disorders Identification Test-Consumption (AUDIT-C). Based on AUDIT-C results, the EHR alerts the MA to give providers a handout on alcohol use and health for patients needing a brief intervention (if AUDIT-C ≥ 3 points women or ≥ 4 men), and/or to ask patients to complete the paper Alcohol Symptom Checklist, which includes 11 questions based on DSM, 5th Edition (DSM-5) AUD criteria (if AUDIT-C 7-12). EHR prompts were also developed to alert providers about the need for a “warm handoff” to an LICSW or to schedule follow-up care to initiate treatment for patients with new AUDs. The EHR also prompts MAs at future visits to ask patients with an AUD diagnosis to complete a “monitoring tool” that includes the AUDIT-C.
2. *Performance monitoring and feedback*. Audit and feedback can be effective for changing health care practices, especially when it is repeated and includes targets [65]. The study team developed several metrics for monitoring and providing weekly feedback to PC clinics and delivery system leaders based on data extracted from the EHR [62]. Details about performance monitoring and feedback are provided in an additional file [see Additional file 1].
3. *External practice coaches provide ongoing front-line support for ~ 6 months*. Practice coaching is also a proven approach to quality improvement in PC [63]. This third strategy is a multi-pronged approach using practice coaches to help overcome stigma, improve knowledge, and enhance perceived importance of alcohol-related care (Table 2), while supporting quality improvement processes. Each of these is described in further detail below.

**Table 2:** Front-line support led by practice coaches in the SPARC trial

Partner with a local implementation team • Initial clinic leadership meeting—to schedule meetings and form local implementation team • Initial local implementation team meetings—two 2-h meetings • Weekly 1-h meetings with local implementation team • Monthly meetings with local implementation team and leaders (behavioral health and PC)	
Trainings • One-hour training for all PC providers and staff together • One-hour PC provider and RN training • One-hour MA training for medical assistants and licensed practical nurses • Learning sessions for PC champions from local implementation teams every 2 weeks by telephone	
Addressing stigma • Ten-minute white board video reframing alcohol and heath by Dr. Mike Evans [67] • Handout reframing alcohol and health (Additional file 2)

##### Practice coaches work with interdisciplinary local implementation teams

During the three-clinic pilot [62], two researchers with previous experience in alcohol-related care completed a practice coaching program through the Dartmouth Institute Microsystem Academy. Elements from this training were incorporated into practice coaching to support clinics implementing SPARC clinical care. Coaching has three phases (Fig. 2):

- During the *usual care phase (1 month prior to preparation)*, the practice coach, PI, and behavioral health leaders have an initial in-person meeting with local clinic leadership to provide an overview of the implementation timeline, guidance for choosing the clinic’s interdisciplinary local implementation team, and set the local meeting schedule. The local implementation team includes an MA, PC provider, and LICSW from each clinic, at a minimum, and if possible a registered nurse (RN), the clinic manager, and the PC clinic medical director.
- *The preparation phase* begins 2 months before each clinic’s randomly assigned launch date for SPARC clinical care (and Behavioral Health Integration). At the start of the preparation phase, practice coaches have two 2-h meetings to introduce local implementation team members to SPARC clinical care, as well as Behavioral Health Integration generally. The goal of this meeting is to build team cohesiveness and engage team members in sharing how providing integrated behavioral health care will benefit their patients and support the clinic’s mission. Practice coaches and the team also develop a deeper understanding of the clinic’s mission, patients, staff, communication practices, and workflows. Subsequently, coaches meet weekly with the local implementation team for the remainder of the preparation phase while they pilot and iteratively adapt the core workflow to fit with the clinic’s local culture, develop job aids and clinical tools, and make communication plans with the rest of the PC clinic. The coach also teaches quality improvement skills to team members.
- *The active implementation phase* begins on the randomly assigned day when the clinic is intended to launch SPARC (and Behavioral Health Integration) clinical care. During active implementation, the coach has weekly meetings with the local implementation team for 3 months, and then every other week meetings for the last month. These plan-do-check-adjust (PDCA) meetings use performance feedback data to help teams identify gaps in SPARC and Behavioral Health Integration clinical care and test solutions. One meeting per month is replaced with a larger “PDCA meeting” with local and/or regional leaders and behavioral health partners to increase sustainability by problem-solving larger systemic issues.
- *The sustainment phase* begins after the 4 months of active implementation have concluded, when clinics are no longer supported by a practice coach. During sustainment, clinics receive monthly performance monitoring and feedback and have quarterly PDCA meetings with Behavioral Health Service leaders.

##### Trainings

In addition to weekly meetings with the local implementation team, practice coaches and/or other SPARC team members lead three 1-h trainings for each PC clinic during the preparatory phase prior to launching SPARC (and Behavioral Health Integration). Details about training content and participants are included in an additional file [see Additional file 1].

##### Addressing stigma

Innovative materials were developed to address stigma during the SPARC pilot, including a patient handout “Alcohol and Health” and a short entertaining video with whiteboard drawing [67]. Both tools reframe unhealthy alcohol use by addressing stereotypes and providing new knowledge about alcohol and health. The handout was designed for PC providers and RNs to use with patients during a brief intervention and includes the following: current scientific views of screening for alcohol and AUDs in general (vs old stereotypes), recommended limits, alcohol-related medical conditions, and symptoms of AUDs. Providers and RNs are trained to offer preventive brief interventions to all patients who “drink regularly” (i.e., AUDIT-C ≥ 3 points women, ≥ 4 men) which is less stigmatized than referring to “positive” screens for risky drinking. The alcohol video is used as part of the initial 1-h training with all PC staff to help all staff understand a shift from an old focus of addressing alcohol use only with patients with recognized AUDs to a broader approach that addresses the entire spectrum of unhealthy alcohol use in PC (from risky drinking to AUDs), including prevention. A link to the video [67] is included in the patient handout.

##### Weekly formative evaluation meetings

Throughout the trial, practice coaches meet weekly with the trial’s principal investigator and the research project manager (who takes detailed notes) for formative evaluation to identify barriers, facilitators, and adaptations necessary for implementation strategies to be successful. Each practice coach reports on experiences in the field that week, by clinic, and issues are identified to discuss in the weekly Behavioral Health Integration operations team meeting.

### Methods for evaluating the impact of the SPARC intervention

#### Study design and sample

**Fig. 3:**
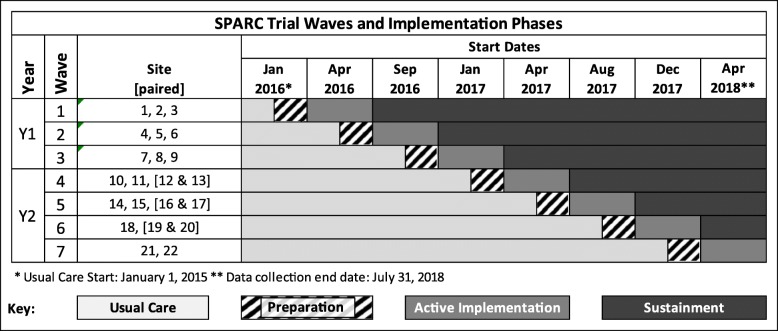
SPARC stepped-wedge pragmatic clinical trial design. *Usual care start: January 1, 2015. **Data collection end date: July 31, 2018. Twenty-two clinics (with three paired to create 19 randomized clinical sites total) were randomized across seven waves with stratification (three waves year 1 and four waves years 2–3). Clinics in square bracket are paired as one site

To evaluate the impact of the SPARC intervention on sustained receipt of evidence-based alcohol-related care, we are conducting a pragmatic stepped-wedge trial in 22 KPWA PC clinics (Fig. 3). A stepped-wedge approach [68] was chosen so that all clinics would eventually receive the intervention and because providing practice coaches for more than four clinics at a time was not feasible. The trial has seven waves staggered by 4 months, such that the final 2 months of the active implementation phase of one wave overlaps with the preparation phase of the next wave. The sample for the study is patients 18 years of age and older who have a PC visit in one of the 22 participating clinics during the study period.

#### Randomization

Stratified randomization was used, with 9 clinics randomized in year 1 (three sites in each of three waves), and the remaining 13 clinics randomized in years 2–3 (Fig. 3). Clinics randomized in year 1 are referred to as Y1 sites and those randomized in years 2–3 as Y2 sites. The random assignment to study wave within Y1 and Y2 was generated using a computer-generated list of random numbers by the study biostatistician after all sites were recruited. An additional file describes our randomization scheme and rationale in detail [see Additional file 1].

##### Four phases of the SPARC trial

Figure 2 shows the four phases of implementation in each clinic. As above, each clinic has approximately 6 months of support from practice coaches, including up to 8 weeks in the “preparation” phase, before the official launch date of SPARC/Behavioral Health Integration, followed by the 4-month “active implementation” phase after the launch date. During the preparation phase, the local implementation team designs and pilots the workflow, with EHR prompts often activated for individuals involved in the piloting. The “launch date” is predetermined as the date when the clinic is intended to activate EHR clinical decision support for all its providers. The stepped-wedge design means that the clinics have varying amounts of time in the usual care phase—before the preparation phase begins, as well as in the sustainment phase—after the active implementation phase ends (Fig. 3).

#### Quantitative evaluation

##### Data collection

All data used to identify the sample, quantitative outcomes, and covariates for this trial are obtained from the Epic EHR and insurance claims. Discrete EHR data and dates are obtained for screenings (e.g., AUDIT-C), assessments (e.g., Alcohol Symptom Checklist), ICD-9 and ICD-10 codes, procedure codes (e.g., V and Z codes used to document brief intervention), medications (e.g., naltrexone), and KPWA utilization inside and outside PC including location (e.g., PC visits to each clinic, visits to LICSWs or specialty mental health clinics). KPWA does not have any internal specialty addiction treatment programs, but data on utilization of specialty addiction treatment in the community will be available from insurance claims.

In addition, natural language processing (NLP) will be used to identify brief interventions documented as templated-free text in the EHR. To identify all templates used to document alcohol-related advice or counseling before and/or after SPARC implementation, including templates made by individual providers as well as those developed by a KPWA quality and clinical improvement office, NLP is used to identify text documentation that includes any keywords and their abbreviations (e.g., alcohol, EtOH, and drink). Identified text that repeats is reviewed by research staff to identify documentation that indicates alcohol-related counseling or advice to change drinking. Templated text identified as brief intervention includes documentation summarized in progress notes and after-visit summaries.

##### Outcome measures

Table 3 outlines the two primary outcomes as well as intermediary measures used to derive the outcomes. Below, we outline the rationale for each main outcome.

**Table 3:** SPARC trial primary, secondary, and other outcomes from EHR and claims data

Category	Measure	Description
Primary outcomes		
Prevention	Alcohol Brief Intervention	Indicator for whether a patient had a brief intervention documented in the EHR* on the day of, or in the 14 days following a PC visit, and had a positive alcohol screen on the day of the visit or in the prior 365 days*
Treatment	Treatment for Newly Diagnosed AUD (NCQA)	Indicator for whether a patient had a new AUD diagnosis* and initiated and engaged in AUD treatment*
Intermediate outcomes		
Prevention	Alcohol screening documented	Indicator for whether a patient had AUDIT-C screening documented in the EHR on the day of the visit or in the prior 365 days
Prevention	Positive alcohol screen	Indicator for whether a patient screened positive on the AUDIT-C (3–12 women and 4–12 men)
Prevention	High-positive alcohol screen	Indicator for whether a patient had a high-positive AUDIT-C score (7–12 points)
Assessment	Assessed for DSM-5 AUD symptoms	Indicator for whether a patient with a high-positive screen completed an AUD Symptom Checklist on the day of the visit or in the prior 365 days
Identification	Past-year AUD diagnosis	Indicator for whether a patient had an AUD diagnosis defined as an ICD code for an AUD diagnosis per NCQA anywhere in or outside KPWA (e.g. includes claims) on the day of the PC visit or in the prior 365 days
Identification	New AUD diagnosis	Indicator that a “past-year AUD diagnosis” (defined immediately above) was new on the day of the PC visit, based on no AUD diagnosis in the prior 365 days
Treatment	Initiation of AUD treatment (NCQA)	Indicator for whether a patient received a “new AUD diagnosis” (defined above) and initiated AUD treatment in the following 14 days, per HEDIS ICD codes
Treatment	Engagement in AUD treatment (NCQA)	Indicator for whether a patient who initiated AUD treatment (defined above) had another 2 treatment visits in the following 30 days after initiation (“engagement”) per HEDIS ICD codes

^*^Definitions based on intermediate outcomes

*EHR* electronic health record, *HEDIS* Healthcare Effectiveness Data and Information Set, *NCQA* US National Committee for Quality Assurance

*Alcohol SBI*. The primary measure of alcohol SBI requires that a patient who has a PC visit has screened positive for unhealthy alcohol use on the day of the visit or in the past year (AUDIT-C ≥ 3 for women and ≥ 4 for men) and has a brief intervention documented on the day of the visit or in the following 14 days [48]. Our indicator of brief intervention is a composite measure based on two data sources: NLP and ICD codes. Patients are considered to have a brief intervention on a certain day if NLP indicates they had documentation of brief intervention with a template on that day (as described above under data collection) and/or if the PC visit is coded with a V or Z code for brief intervention, from ICD-9 and ICD-10 systems, respectively.

The NCQA released a new (2018) HEDIS performance measure for alcohol SBI with a 2-month window for follow-up, in contrast to the 14-day window of our primary outcome, so this will be evaluated as a secondary outcome [69]. Changes over the four phases of implementation in the prevalence of alcohol screening and positive AUDIT-C screens will be described as intermediary measures.

*AUD treatment.* The primary measure of AUD treatment is whether patients with a new AUD diagnosis initiate and engage in treatment for AUD. The definitions for initiation and engagement are based on ICD-9 or ICD-10 codes and timeframes used in NCQA’s alcohol or drug (AOD) HEDIS measures [69], a commonly used definition of AUD treatment. However, the definition used for a “new AUD diagnosis” for the primary outcome differs from NCQA’s measure: instead of referring to the first AUD diagnosis *in each calendar* year with no AUD diagnosis in the prior 60 days, the measure of new AUD diagnoses used in this trial requires a 1-year “look-back” period with no AUD diagnosis. The primary measure requires a face-to-face visit coded with an AUD diagnosis within 14 days of a new diagnosis (consistent with the HEDIS AOD “initiation” measure during most of the trial), and two more visits coded with AUD diagnoses in the 30 days after initiation (consistent with HEDIS “engagement” visits). Because it often takes longer than 14 days to wait for a follow-up PC appointment, making it difficult for patients with AUD to have three visits in PC in 44 days, and because the HEDIS AOD measure changed to include telephone visits starting in 2018, sensitivity analyses will evaluate whether our findings are sensitive to timeframe and exclusion of telephone visits, by allowing initiation in 30 days, engagement in 60 days, and inclusion of telephone visits.

The HEDIS AOD measures might categorize health care visits as AUD treatment when in fact they are not because clinicians can appropriately code visits with an AUD diagnosis to indicate their care is *complicated by* AUDs rather than reflecting *treatment of* AUD. Therefore, to estimate how often patients are likely to be receiving behavioral or medication treatments for AUDs, secondary, more stringent measures of AUD treatment will consider visits coded for AUDs to indicate initiation and engagement of AUD treatment only if they are visits to a behavioral health provider or visits in which AUD medications are prescribed in the 44 days after the new diagnosis, or a visit to specialty addiction treatment outside KPWA (Table 3).

*Samples and time intervals*. A population-based denominator is used in all analyses as the least-biased denominator because clinical site implementation of the SPARC and Behavioral Health Integration interventions is expected to change the proportion and characteristics of patients screened, the proportion who screen positive for unhealthy alcohol use, and the proportion and characteristics of those diagnosed with AUDs. Unless otherwise specified, the sample for each measure is the set of patients who have a PC visit to a PC clinical site for any reason during each time interval used in analyses. Time intervals used in analyses are typically 4 weeks (i.e., 28-day intervals before and after each clinic’s specified launch date). This interval is selected to provide adequate numbers of outcomes per interval [62]. Specifically, primary analyses will compare the monthly (28-day) proportion of patients with each outcome in the pre- versus post-implementation periods.

#### Statistical analysis

Main analyses of the trial compare two primary outcomes—alcohol SBI and AUD treatment—among PC patients seen in the participating PC clinics before and after the randomized SPARC launch (Fig. 2 and Table 4). Primary analyses will compare the monthly outcome rates of the two primary outcomes before and after launch. Secondary analyses will also compare primary outcomes across other study phases (Fig. 2 and Table 4).

**Table 4:** The primary analysis

Primary analyses compare months before vs. months after the assigned launch date (usual care + preparation phases vs. active implementation + sustainment phases). Secondary analyses a. Usual care vs. active implementation b. Usual care vs. sustainment c. Active implementation vs. sustainment	

Analyses will follow the general framework for analyzing data from a stepped-wedge trial [70, 71]. The intervals used in analyses are 28-day periods (“month” hereafter) before and after the launch date for each clinic. Specifically, we will model indicator variables for the primary outcomes monthly (e.g., indicator for whether each patient who had a PC visit that month received alcohol SBI or a new AUD diagnosis and treatment) using the following logistic mixed-effect model:

$$ logit\ P\left({Y}_{ijm}=1\right)=\alpha +\beta {Int}_{jm}+\gamma {S}_j+f(cm)+{b}_j+{u}_i, $$

where *Y*_*ijm*_ is the outcome for person *i* who visited site *j* in month *m*. The term *Int*_*jm*_ is an indicator variable for whether the visit month was before or after the randomly assigned SPARC launch date for that site (i.e., if the site was in the usual care or preparation vs. active implementation or sustainment phases; Fig. 1 and Table 4). Following intention-to-treat principles, unless otherwise specified, phases will be defined based on official randomized launch dates, when active implementation was planned to start, rather than the actual date when the site began implementing SPARC (if implementation was delayed). The term *S*_*j*_ is an indicator for whether site *j* was a Y2 versus a Y1 site (stratification variable), which accounts for possible differences in outcomes across these two groups of sites, and *f*(*cm*) is a pre-specified function of calendar month of the study when the PC visit occurred (1–31) to account for the potential for a secular trend in the outcome rates over time (January 2015–July 2018). We plan to model *f*(*cm*) using indicator variables for seven 4-month periods. Additionally,${u}_i\sim N\left(0,{\tau}_u^2\right)$ and ${b}_j\sim N\left(0,{\tau}_b^2\right)$ are person- and site-level random intercepts to account for correlation of outcomes from the same individual over multiple months and of individuals from the same site, respectively. The primary analysis (Table 4) will be a two-sided Wald test (at the 0.05 level) of the coefficient *β*, which denotes the log odds ratio comparing the monthly outcome rate in the post period to the monthly outcome rate in the pre-period. We will also calculate 95% Wald confidence intervals (95% CI) for *β*. Secondary analyses of changes across all four phases will be accomplished by replacing the *Int*_*jm*_ term with a categorical variable for whether the month of the PC visit was in the usual care, active implementation, or sustainment phase and for testing the relevant contrast.

Secondary analyses, parallel to the primary analyses, will assess each intermediate outcome, as explanatory analyses, in the pre- versus post-implementation periods. If increases in alcohol screening and AUD assessment (Table 3) are observed, secondary patient-level analyses will evaluate whether screening is associated with increased brief interventions, and whether completing an Alcohol Symptom Checklist is associated with increased new AUD diagnoses. Additional secondary analyses and sensitivity analyses are described in an additional file [see Additional file 1].

#### Statistical power

With 19 sites, seven study waves (with number of sites per wave described above), and 4 months between launch dates across waves, and assuming an average of 1205 patients seen per site per month (based on baseline data obtained at the time of the grant proposal for the trial), we will have 80% (90%) power to detect an increase in brief intervention rates of 7.1 (8.2) per 10,000 patients seen and an increase in treatment engagement of 2.6 (3.1) per 10,000 patients seen. Calculations, which were based on a two-sided test and a type 1 error rate of 0.05 and used the method of Hussey and Hughes (2007), assumed the following usual care rates for the main study outcomes: 34.2 per 10,000 patients seen for the brief intervention outcome (BI; 0.342% = 19% screened × 36% screened positive × 5% brief intervention) and 3.9 per 10,000 for treatment initiation and engagement (0.039% = 1.26% newly diagnosed × 37.5% initiating treatment × 8.2% engaged). We further assumed a value for the intraclass correlation coefficient of 0.001, based on baseline data from the included sites.

#### Qualitative evaluation

##### Data collection

Several sources of qualitative data are routinely collected during the trial. Detailed typed minutes are taken during weekly operations meetings. In addition, detailed typed notes are taken of discussions during weekly formative evaluation meetings, using a spreadsheet to document the meeting date, clinic site, and a summary of practice coaches’ descriptions of the current state of implementation at that site, including barriers and facilitators and any resulting implementation adaptations. All names are omitted from notes to protect confidentiality (only clinical roles are recorded).

##### Qualitative analyses

We will use a rapid assessment process that was developed for this project, building on prior methods [72], to summarize findings regarding changes in the health system during the trial, adaptations to SPARC implementation strategies, and barriers and facilitators encountered during implementation. These analyses will be guided by Greenhalgh’s conceptual framework for dissemination of innovations [40, 41]. Details about the process for conducting these analyses and linking results to quantitative site performance data are included in an additional file [see Additional file 1]).

### Trial status

This protocol reflects a proposal reviewed by the Agency for Health Research and Quality (AHRQ) in 2013 and funded in 2014. At the time of submission, the research team is implementing care in the final wave of PC clinics (Fig. 3).

## Discussion

If implementation succeeds at implementing SBI and increasing diagnosis and treatment of AUD in the SPARC trial, findings of this trial will help other systems wishing to implement alcohol-related care in PC. This study will create a roadmap and make widely available online tools to help other healthcare organizations improve the quality of alcohol-related care. We will disseminate findings via AHRQ’s online site for integrating behavioral health and PC [73] as well as via our own research website.

### Strengths and limitations

A significant strength of this pragmatic trial is that it evaluates a range of outcomes using secondary data. This strategy does not rely on patient interviews, which yield smaller, biased samples and impose significant recruitment and consent burdens and survey costs. However, this design introduces other limitations. Primary outcomes are defined based on EHR documentation and assumed to be absent if EHR documentation is lacking. We use an NLP measure of brief intervention based on templates because clinical leaders wanted to focus providers on offering brief counseling or advice rather than standardized documentation by “clicking a box” in the EHR [50]. However, resources were insufficient to develop and validate an NLP measure of any alcohol-related advice or counseling in the EHR. We therefore use text from repeated EHR templates identified with NLP and coded as brief intervention. Although most clinicians use or develop templates to speed frequent documentation, this approach to measurement likely underestimates brief intervention. Moreover, documentation does not reflect the quality of the discussion about alcohol, and whether the discussion included explicit advice to cut down or abstain [74]. Secondary data are being sought to use a 2017 Washington State Health Alliance survey [75] that includes KPWA and included a previously used patient-report measure of brief intervention [43], to compare rates of patient-reported alcohol-related advice in KPWA sites surveyed before, during, and after SPARC implementation. In addition, the primary outcome measure for AUD treatment is defined based on ICD codes and time frames used for NCQA’s HEDIS measures for AUD treatment. Documentation of an AUD ICD code is assumed to represent AUD treatment. We use this definition for AUD treatment to maximize relevance to healthcare systems. However, documentation of an ICD codes for AUD is not an indicator of AUD treatment, as AUD ICD-9 or ICD-10 codes can be used in billing for a medical visit any time the condition is relevant to care (e.g., if a condition impacts care of another condition). Therefore, secondary analyses will assess a more stringent definition of AUD treatment [43, 74]. Moreover, while patients may underreport their alcohol use or their symptoms in clinical care, many patients do not [62]. Finally, this study was limited to adults visiting family medicine clinics. Future research is needed to determine the optimal approach to implementing alcohol-related care for adolescents less than 18 years old.

### Conclusion

Alcohol is the third greatest cause of disability and death in the USA [5, 76, 77], but prevention and treatment of unhealthy alcohol use has not historically been integrated into routine medical care. With evidence-based interventions for unhealthy alcohol use available, experts now realize the crucial step is providing screening and treatment in routine medical settings [78–83]. Developing an effective set of strategies to implement and sustain evidence-based alcohol-related care, testing them in a pragmatic trial, and disseminating the results widely, has the potential to transform healthcare practice to address the full spectrum of unhealthy alcohol use in PC settings.

## Additional file

Additional file 1: Additional details about the conduct and analysis of the SPARC Trial. (DOCX 41 kb)

Additional file 2: Alcohol and Health handout that reframes unhealthy alcohol use by addressing stereotypes. (DOCX 41 kb)

## Abbreviations

AOD: Alcohol or drug
AUDIT-C: Alcohol use Disorders Identification Test-Consumption
AUDs: Alcohol use disorders
BI: Brief intervention
CSSRS: Columbia Suicide Severity Rating Scale
DSM-5: *Diagnostic and Statistical Manual*, 5th edition
DUD: Drug use disorder
EHR: Electronic health record
ICD-9: International Classification of Diseases codes, 9th edition
KPWA: Kaiser Permanente Washington
KPWHRI: Kaiser Permanente Washington Health Research Institute
LICSW: Licensed independent clinical social worker
MA: Medical assistant
NCQA: US National Committee for Quality Assurance
NLP: Natural language processing
PC: Primary care
PDCA: Plan-do-check-adjust
PHQ-2: Two-item Patient Health Questionnaire depression screen
PHQ-9: Nine-item Patient Health Questionnaire depression screen
RN: Registered nurse
SAMHSA: Substance Abuse and Mental Health Services Administration
SBI: Screening and brief intervention
SPARC: Sustained Patient-centered Alcohol-related Care
VA: Veterans Affairs
