# Supplementary material for: Study protocol: a cluster-randomized trial implementing Sustained Patient-centered Alcohol-related Care (SPARC trial)
Source: Implement Sci. 2018 Aug 6;13:108. doi: 10.1186/s13012-018-0795-9 (PMC6080376; doi:10.1186/s13012-018-0795-9)
Supplement: Supplementary file 1 — Additional details about the conduct and analysis of the SPARC Trial. (DOCX 41 kb) [file 13012_2018_795_MOESM1_ESM.docx]

**Additional File 1**

**Additional details about the research-operations partnership**

The SPARC Trial is a partnership between researchers at Kaiser Permanente Washington Health Research Institute (KPWHRI) and Kaiser Permanente Washington (KPWA) clinical leaders. SPARC built on an existing but relatively new partnership between KPWHRI and KPWA Behavioral Health Services. Weekly “Behavioral Health Integration” operations meetings include research leaders and staff, behavioral health clinical leaders from addictions and consultative psychiatry as well as social work, and practice coaches funded by the research grant. This meeting is used to plan implementation activities (e.g., trainings, communications with the health system) and address challenges identified by the practice coaches and local implementation teams at the primary care (PC) sites (e.g., modifications to electronic health record [EHR] tools and performance metrics). An additional leadership meeting every 2-4 weeks includes the above research and behavioral health clinical leaders, and the medical director of behavioral health. PC leaders were invited to the Behavioral Health Integration operations and leadership meetings when available. In addition, the research grant supports a programmer to collaborate on development, reporting and refinement of performance monitoring during the trial, as well as an EHR programmer who meets weekly with researchers, clinical leaders and a practice coach to develop clinical decision support tools. The KPWA Behavioral Health Service supports their clinical leaders’ involvement in SPARC, as well as 10% time for the grant's principal investigator, a general internist. The KPWHRI research team designed and initially provided all Behavioral Health Integration forms and alcohol-related handouts in partnership with KPWA clinical leaders during the pilot, and provides support for the transition of production and ordering of forms to the KPWA clinical system. Eventual transfer of performance monitoring and feedback from research to delivery system leaders is planned.

**Additional details about the health system addition of integrated behavioral health clinicians trained in managing substance use disorders**

As part of the shift of the role of social workers in PC from that of medical social workers to integrated behavioral health clinicians, all social workers were required to be licensed independent clinical social workers (LICSWs). LICSWs initially receive 2-4 hours of training addressing substance use disorders with shared decision-making, with an additional 4 training hours every 6 months (e.g. regarding motivational and cognitive behavioral interventions). An EHR registry identifies patients with new substance use disorder diagnoses, and LICSWs are asked to reach out to those patients who are not yet engaged in substance use-related care. Social work leaders hold weekly telephonic supervision meetings to review the registry of patients with substance use disorders and problem-solve barriers to patient engagement. Weekly supervision includes four members of the SPARC team (internist/PI, research psychologist, psychiatrist who leads addictions and consultative psychiatry, and social work manager).

**Additional details about EHR decision support**

EHR decision support was developed to guide screening for, assessing, and managing unhealthy alcohol use (Table 1 in article).^1^ During PC visits, a “pre-visit summary” prompt in the EHR alerts medical assistants (MAs) or other staff who room patients (MAs hereafter) that a patient has not had alcohol screening within the past year with the 3-item Alcohol Use Disorders Identification Test-Consumption (AUDIT-C). MAs ask appropriate patients to complete a self-administered Behavioral Health Integration screening questionnaire. When MAs enter screening answers from the self-administered paper questionnaires into the EHR, the EHR automatically totals scores and alerts MAs to give providers a handout on alcohol use and health for patients needing a brief intervention (AUDIT-C scores 3+ for women, 4+ for men). MAs also ask patients with high-risk AUDIT-C scores (7 or more points) to complete a self-administered Alcohol Symptom Checklist including the 11 criteria for DSM-5 alcohol use disorder (AUD); when these data are entered into the EHR, it automatically totals and interprets the severity of symptom checklist scores to help PC providers assess patient symptoms and guide diagnosis: 0–1 symptoms = no AUD; 2–3 = possible mild AUD; 4–5 = possible moderate AUD; and 6+ = possible severe AUD. If an Alcohol Symptom Checklist is missed during a clinic visit, MAs are alerted to have the patient complete an Alcohol Symptom Checklist at the next PC visit. An EHR prompt was developed to alert providers about the need for a “warm handoff” to an LICSW or to schedule follow-up care to initiate treatment for patients with new AUDs. The EHR also prompts MAs during future visits to ask patients who have received an AUD diagnosis to complete a monitoring tool that includes the AUDIT-C.

**Additional details about performance monitoring and feedback**

During a 3-clinic pilot,^2^ the study team developed several metrics for monitoring and providing feedback to PC clinics and delivery system leaders based on data extracted from the EHR. Feedback provided to clinics includes the weekly and monthly proportion of PC patients screened for unhealthy alcohol use. For those who screen positive for high-risk unhealthy alcohol use (AUDIT-C≥7), the feedback includes the proportion assessed with the DSM-5 Alcohol Symptom Checklist. Clinics receive weekly metrics reflecting the proportions of patients screened and assessed with the goal of screening patients annually (targets for both are 80%). Providers are not given feedback regarding brief intervention due to the challenges of measuring BI documentation in “real time”. Though other health systems, such as the VA, allow documentation by “checking a box” that sends standardized text to the progress note that can be easily retrieved, KPWA clinical leaders had concerns that this would result in greater focus on documentation than counseling.^3, 4^ Instead, clinical leaders encouraged providers to learn to conduct comfortable, high quality brief intervention, and document it in whatever ways felt comfortable. Standardized tools are available to assist documentation, but are not required.^3, 4^ Measures for monitoring and feedback on rates of AUD diagnosis and treatment were under development at the time of Y1 randomization to supplement the measure on DSM-5 assessment with the Symptom Checklist.

**Additional details about trainings conducted by external practice coaches**

In addition to weekly meetings with the local implementation team, during the preparatory phase prior to launching SPARC (and Behavioral Health Integration), practice coaches and/or other SPARC team members lead three 1-hour trainings for each PC clinic. An initial one-hour training for all PC clinic staff, including PC providers, allows the local implementation team to share piloting stories and their experience with the new work and give their colleagues an overview and rationale for Behavioral Health Integration. A training for PC providers and RNs reviews screening and assessment forms, workflow, coding and diagnosing AUDs, conducting alcohol brief interventions, and offering treatment options for AUD. An MA training focuses on learning scripting for introducing the self-administered Behavioral Health Integration screening questionnaire to patients; workflow for entering data from paper questionnaires into the EHR during rooming, and when to alert PC providers and LICSWs about positive screens. Learning sessions were planned every other week by telephone provide a time for PC clinicians from multiple local implementation teams to learn from each other and receive further training.

**Additional details about the randomization scheme**

The randomization scheme addressed four practical concerns of health system leaders while maintaining a rigorous evaluation of the SPARC implementation intervention (Figure 3 in article). First, health system leaders asked that they be allowed to choose the first 9 clinics that started SPARC/Behavioral Health Integration year 1 (first 3 waves). As a result, a stratified randomization was used, and health system leaders identified 9 clinics that would be randomized in Year 1 (3 sites in each of 3 waves), with the remaining 13 clinics randomized in Years 2-3. Clinics randomized in Year 1 are referred to as Y1 sites and those randomized in Years 2-3 as Y2 sites. Second, in Y2, six clinics were grouped into three pairs of clinics at the request of leaders and to facilitate coaching because they were located far from the KPWHRI offices (1-4 hours travel time). This change resulted in 19 total clinical sites that were randomized. Third, one of the Y2 sites (which had two clinics) needed to be in a wave with only one other site due to travel time to the sites. Fourth, the final wave needed to have only two sites to address leaders’ request that all sites implement as soon as possible.

To address the above issues, randomization of the 19 clinical sites (including 3 sites that were pairs of clinics as above) proceeded as follows. First, per stratification (Y1 vs. Y2), the nine sites selected for Y1 were randomly assigned to begin implementation on one of 9 dates in the three Y1 waves (three sites per wave staggered by 1 week). Second, the remaining 10 clinical sites to be randomized in Y2 would be randomly assigned to 4 Y2 waves: two 2-site waves and two 3-site waves. Because the last wave needed to be a 2-site wave, one of the other 3 waves in Y2 was randomly selected to be the other 2-site wave. We then developed a randomization scheme that ensured that each Y2 site had the same probability of being assigned to each of the 4 Y2 waves, given the constraints above. The random assignment to study wave within Y1 and Y2 was generated using a computer-generated list of random numbers by the study biostatistician after all sites were recruited.

**Additional details about the secondary analyses and sensitivity analyses**

Additional secondary analyses are designed to estimate the sensitivity of findings regarding AUD treatment to the definition of AUD treatment used (National Committee for Quality Assurance [NCQA] Healthcare Effectiveness Data and Information Set [HEDIS] International Classification of Diseases [ICD] codes and timeframes). These secondary analyses vary time frames for initiation and engagement, settings (face-to-face vs. also telephone or video), and limiting AUD treatment to: AUD treatment from a behavioral health specialist or specialty addictions treatment program, or AUD medications.

Additionally, to examine the potential for time-varying effects of the SPARC implementation intervention, the ${Int}_{jm}$ term will be expanded to allow for separate effects at each month from 4 months prior through up to 12 months after the active implementation start date. We also plan to explore whether the effect of the SPARC implementation differs in Y1 sites versus Y2 sites by including an interaction term between randomization year and the intervention effect (i.e., between $S_{j}$ and $Int_{jm}$).

Estimates of intervention effects under the stepped-wedge design may be subject to confounding due to time trends. Confounding due to time trends is addressed in this study via the function of calendar time $f(cm)$. We therefore plan to conduct sensitivity analyses including finer specifications of the time trend $f(cm)$ in months (instead of 4-month intervals) and interaction terms between $S_{j}$ (stratification variable) and $f(cm)$. If we find that several sites did not begin implementing SPARC on the planned launch date, we will also consider a “per-protocol” analysis for our primary outcomes that uses the actual implementation start date (when EHR clinical prompts were “turned on” for all PC providers.

**Additional Details About the Qualitative Analyses**

We will conduct a rapid assessment process that was developed for this project, building on prior methods,^5^ to summarize findings regarding: changes in the health system during the trial; adaptations to SPARC implementation strategies; and barriers and facilitators encountered during implementation. These analyses will be guided by Greenhalgh’s conceptual framework for dissemination of innovations.^6, 7^ To accomplish this process efficiently, a 4-hour debrief will initially be used to identify major issues by implementation wave based on collective team memory. This debrief will include all SPARC team members who attended weekly operations meetings, as well as the Co-PI and an implementation researcher not involved in implementation. The goal of the debrief is to create an outline (e.g. template) of major themes and sub-themes regarding: health system changes, SPARC implementation adaptions, and barriers and facilitators, focusing on those of potential relevance for future implementation efforts that occurred in the sites in each successive wave. After the debrief, two readers will review all notes from weekly operations and formative evaluation meetings to identify potentially important themes and sub-themes omitted, adding those to the template. Subsequently, themes in the template will be categorized into Greenhalgh’s conceptual framework for dissemination of innovations, which was used in design of the implementation intervention and previously used to evaluate implementation of alcohol SBI in the VA.^6, 7^ Major domains of the Greenhalgh Model used to code the qualitative themes will include: components of the User System (health system antecedents for innovation, system readiness for innovation, adopters, assimilations, implementation processes, consequences of implementation, implications of consequences for the User System), and an Innovation System (the Resource System, Knowledge Purveyors, Change Agency, their Communication and Influence, and the Innovation), the Linkage of the User and Innovation Systems, and the Outer Context (sociopolitical climate, interorganizational norm-setting). The entire SPARC study team (those involved in debrief) will meet to review and edit the outlines for each domain and finalize themes and identify prototypical content within each theme for presentation.^8^ A final debrief will be held, after quantitative findings are complete, to review each site’s performance and link it to the qualitative findings in a secondary manuscript.

**References in Additional File 1**

1. Damiani G, Pinnarelli L, Colosimo SC, Almiento R, Sicuro L, Galasso R, Sommella L, Ricciardi W. The effectiveness of computerized clinical guidelines in the process of care: a systematic review. BMC Health Services Research. 2010;10:2.

2. Bobb JF, Lee AK, Lapham GT, Oliver M, Ludman E, Achtmeyer C, Parrish R, Caldeiro RM, Lozano P, Richards JE, Bradley KA. Evaluation of a Pilot Implementation to Integrate Alcohol-Related Care within Primary Care. Int J Environ Res Public Health. 2017;14. PMCID: PMC5615567.

3. Chavez LJ, Williams EC, Lapham GT, Rubinsky AD, Kivlahan DR, Bradley KA. Changes in Patient-Reported Alcohol-Related Advice Following Veterans Health Administration Implementation of Brief Alcohol Interventions. J Stud Alcohol Drugs. 2016;77:500-8. PMCID: PMC4869906.

4. Berger D, Lapham GT, Shortreed SM, Hawkins EJ, Rubinsky AD, Williams EC, Achtmeyer CE, Kivlahan DR, Bradley KA. Increased Rates of Documented Alcohol Counseling in Primary Care: More Counseling or Just More Documentation? J Gen Intern Med. 2018;33:268-74. PMCID: PMC5834950.

5. Beebe J. Rapid Assessment Process: An Introduction: Altmira Press; 2001.

6. Greenhalgh T, Robert G, Macfarlane F, Bate P, Kyriakidou O. Diffusion of innovations in service organizations: systematic review and recommendations. Milbank Q. 2004;82:581-629. PMCID: PMC2690184.

7. Williams EC, Achtmeyer CE, Young JP, Rittmueller SE, Ludman EJ, Lapham GT, Lee AK, Chavez LJ, Berger D, Bradley KA. Local Implementation of Alcohol Screening and Brief Intervention at Five Veterans Health Administration Primary Care Clinics: Perspectives of Clinical and Administrative Staff. J Subst Abuse Treat. 2016;60:27-35.

8. Morse JM, Barrett M, Mayan M, Olson K, Spiers J. Verification Strategies for Establishing Reliability and Validity in Qualitative Research. International Journal of Qualitative Methods. 2002;1:13-22.
